# The impact of leg position on muscle blood flow and oxygenation during low-intensity rhythmic plantarflexion exercise

**DOI:** 10.1007/s00421-022-05117-9

**Published:** 2023-01-16

**Authors:** Kyohei Marume, Hendrik Mugele, Ryo Ueno, Sachin B. Amin, Heru Syarli Lesmana, Carmen Possnig, Alexander B. Hansen, Lydia L. Simpson, Justin S. Lawley

**Affiliations:** 1 Department of Sport Science, University of Innsbruck, Innsbruck, Austria; 2 Institute of Mountain Emergency Medicine, Eurac Research, Bolzano, Italy

**Keywords:** Leg position, Resistance training, Muscle oxygenation, Exercise-induced hyperemia, Reactive hyperemia, Flow-mediated dilatation

## Abstract

**Purpose:**

Resistance training (RT) is an effective countermeasure to combat physical deconditioning whereby localized hypoxia within the limb increases metabolic stress eliciting muscle adaptation. The current study sought to examine the influence of gravity on muscle oxygenation (SmO_2_) alongside vascular hemodynamic responses.

**Methods:**

In twelve young healthy adults, an ischemic occlusion test and seven minutes of low-intensity rhythmic plantarflexion exercise were used alongside superficial femoral blood flow and calf near-infrared spectroscopy to assess the microvascular vasodilator response, conduit artery flow-mediated dilation, exercise-induced hyperemia, and SmO_2_ with the leg positioned above or below the heart in a randomized order.

**Results:**

The microvascular vasodilator response, assessed by peak blood flow (798 ± 231 mL/min vs. 1348 ± 290 mL/min; *p* < 0.001) and reperfusion slope 10 s of SmO_2_ after cuff deflation (0.75 ± 0.45%.s-1 vs.2.40 ± 0.94%.s-1; *p* < 0.001), was attenuated with the leg above the heart. This caused a blunted dilatation of the superficial femoral artery (3.0 ± 2.4% vs. 5.2 ± 2.1%; *p* = 0.008). Meanwhile, blood flow area under the curve was comparable (above the heart: 445 ± 147 mL vs. below the heart: 474 ± 118 mL; *p* = 0.55) in both leg positions. During rhythmic exercise, the increase in femoral blood flow was lower in the leg up position (above the heart: 201 ± 94% vs. below the heart: 292 ± 114%; *p* = 0.001) and contributed to a lower SmO_2_ (above the heart: 41 ± 18% vs. below the heart 67 ± 5%; *p* < 0.001).

**Conclusion:**

Positioning the leg above the heart results in attenuated peak vascular dilator response and exercise-induced hyperemia that coincided with a lower SmO_2_ during low-intensity plantarflexion exercise.

## Introduction

Prevention of physical deconditioning is important to prolong the healthy lifespan in an aging society. Physical deconditioning is induced by periods of musculoskeletal disuse, such as sedentary behavior and bed rest, with the greatest atrophy occurring within the lower limb muscles compared to the trunk muscles (LeBlanc et al. 1992). An effective countermeasure to limit the loss in muscle strength and exercise capacity during periods of deconditioning is resistance training (Lopez et al. 2018; Bamman et al. 1997).

While it is widely recognized that mechanical loading of the skeletal muscle is the primary factor driving hypertrophy with resistance training (Adams and Bamman 2012), recent evidence suggests that local skeletal muscle metabolic factors are also important (Schoenfeld 2010, 2013; Pearson and Hussain 2015; Scott et al. 2014). For example, local hypoxia has mechanistic links to several molecular signaling pathways for protein synthesis and vascular growth (Scott et al. 2014) and has therefore lead to its manipulation via techniques such as blood flow restriction (BFR) or systemic hypoxia (Schoenfeld 2010, 2013; Pearson and Hussain 2015; Scott et al. 2014). Indeed, the use of BFR during resistance exercise has become a popular training method whereby several investigations have shown lower skeletal muscle oxygenation during exercise (Killinger et al. 2020) and greater improvements in muscle hypertrophy and strength despite low mechanical loads (20–40% of maximum voluntary contraction [MVC]) in both clinical (Ohta et al. 2003) and athletic (Yamanaka et al. 2012) populations. Morover, an emerging, albeit limted set of studies (Manimmanakorn et al. 2013), has highlighted the potential benefits of intermittent hypoxic resistance training. While both BFR and systemic hypoxia via inhalation of low inspired oxygen fractions (simulated altitude) may manipulate skeletal muscle oxygenation, their widespread implementation is hindered by the need for technical equipment, experienced personnel and potential safety concerns.

An alternative simplified approach to manipulate local skeletal muscle oxygenation during resistance exercise may be to position the limb above heart level. Previous independent studies have observed that positioning the limb above the heart causes a lower tissue oxygenation of the tibialis anterior during dorsiflexion exercise using near-infrared spectroscopy (NIRS) (Tachi et al. 2004) and causes a reduced exercise-induced hyperemia in the forearm (Tschakovsky et al. 1996; Nådland et al. 2009). Therefore, the aim of the current investigation was to confirm the concept that decreasing the hydrostatic gradient above heart level causes a reduction in steady-state muscle oxygenation, which is due in part, to an impaired microvascular dilator response (e.g., change in peak blood flow after cuff deflation) and thus attenuated local skeletal muscle oxygen delivery (i.e., muscle blood flow). We hypothesized that positioning the leg above the heart position would attenuate exercise-induced hyperemia and macro- and microvascular dilator response, and also a lower muscle oxygenation during exercise (SmO_2_).

## Methods

### Participants

Twelve healthy participants (nine males and three females, mean age of 29 ± 3 years) were recruited. Their mean height, weight, body mass index, and thigh circumference were 176 ± 8 cm, 70 ± 9 kg, 22 ± 2 kg/m^2^, and 44 ± 14 cm. All subjects were free from known cardiovascular, respiratory, and metabolic disease, and were not taking any prescription or over-the-counter medication at the time of participation. Two participants were current smokers with a Brinkman index of one and two. The menstrual cycle phase was not controlled in the three female participants. Participants attended the laboratory on two occasions: On the first occasion, their maximal voluntary contraction (MVC) of plantarflexion exercise was measured, and on the second occasion, cuff occlusion test (experimental protocol 1) and rhythmic plantarflexion exercise at 10% of MVC (experimental protocol 2) were conducted. Written informed consent was obtained from all participants following detailed verbal explanations of the experimental protocol that included information regarding all potential risks. The study conformed to the standards set out by the Declaration of Helsinki, except for registration in a database, and was approved by the ethics committee of the University of Innsbruck (No. 34/2018).

### Experimental protocol

**Fig. 1:**
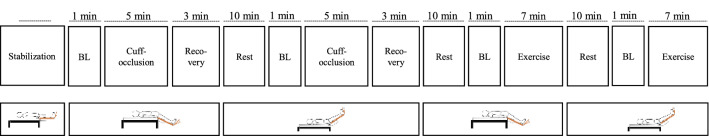
Study protocol timeline. Ischemic test performed by cuff inflation to 220 mmHg for 5 min. Exercise was performed at 10% of maximum voluntary contraction for 7 min.  The order of the leg positions was randomized. BL, Baseline data collection

Participants arrived at the laboratory having abstained from alcohol, caffeine, smoking, and exercise for 12 h and having consumed a light meal four hours prior to testing (Harris et al. 2010). Experiments were performed at the same time of day (12:00–16:00) in a quiet room with a temperature between 20 and 25 °C. Following instrumentation, participants rested in the supine position with their legs at heart level for 20 min to allow central hemodynamic stabilization. The experimental protocol consisted of an ischemic cuff occlusion test (Experimental protocol 1) to assess microvascular vasodilator responses and flow-mediated dilation (FMD) followed by a steady-state plantarflexion exercise (Experimental protocol 2) to examine metabolic flow coupling were performed (Fig. 1). The ischemic cuff tests were performed first, but all protocols were separated by a minimum of 10 min and femoral diameter and velocity were measured to confirm returned to baseline values. To assess the impact of hydrostatic pressure, both protocols we completed with the legs lowered -30 and raised + 30 degrees relative to the heart. The order was randomly assigned and all measurements were performed on the right leg.

#### Experimental protocol 1

In experimental protocol 1, a pneumatic cuff was placed slightly proximal to the knee and after a stable baseline period (~ 1 min), inflated to supra-systolic pressure (220 mmHg) for five minutes, thereafter pressure was immediately released and the participants were instructed to remain still for a further three minutes (Bleeker et al. 2005). Baseline blood pressure was measured one minute before the start of cuff occlusion. All hemodynamics except blood pressure were measured continuously through the protocol.

#### Experimental protocol 2

**Fig. 2:**
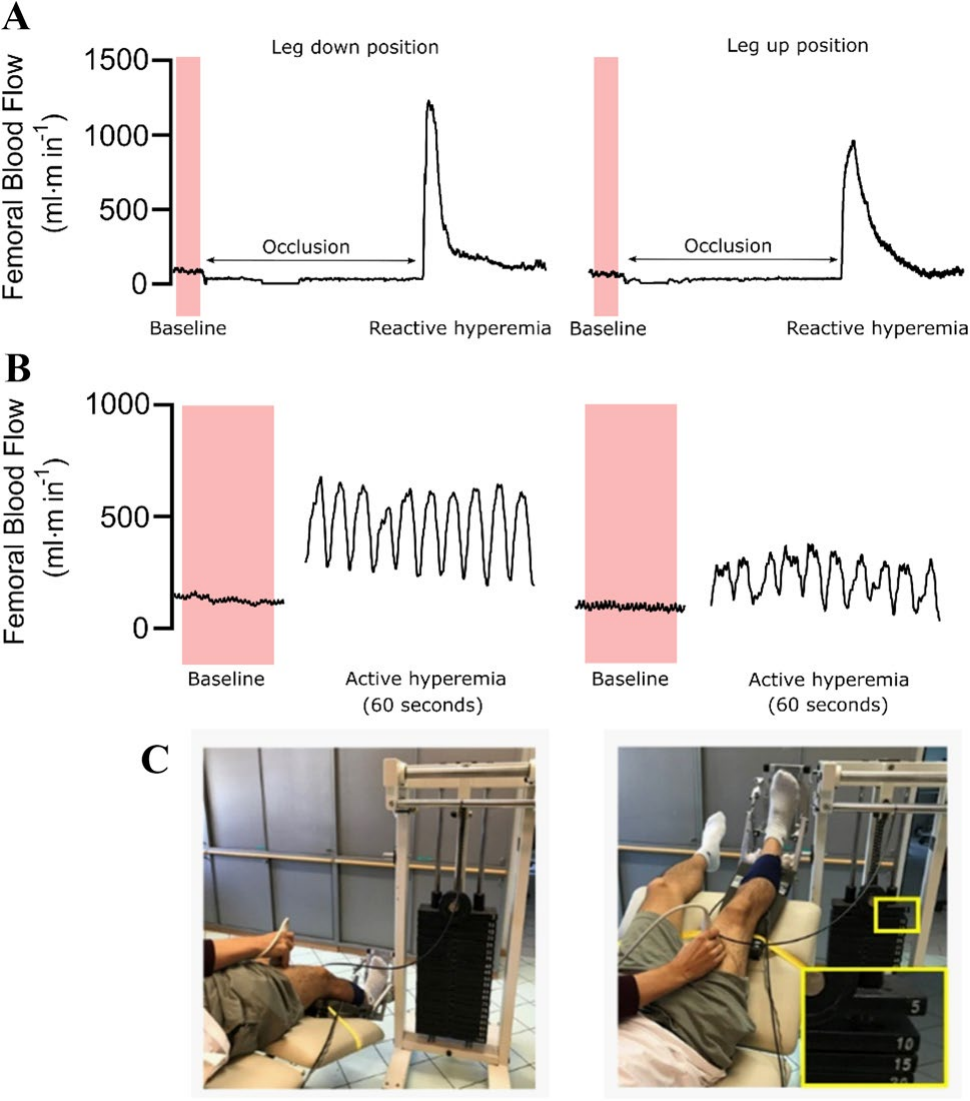
Representative data and equipment for experimental protocol 1 and 2. **a** Representative tracing of femoral blood flow (3 s smoothing window) at baseline, during 5 min of occlusion and over 3 min post cuff release in the leg above the heart and leg below the heart position in one individual. **b**) Raw data for femoral blood flow at rest (baseline) and over the final 10 plantarflexion contraction/relaxation cycles in the leg above the heart and leg below the heart position from the same individual. **c** Custom-made ergometer for plantarflexion exercise

In experimental protocol 2, participants performed rhythmic plantarflexion exercise at 10% of MVC using a custom-made ergometer. Briefly, a footplate was connected to a weight plate machine (Twuwsen, Kranenburg, Germany) to provide smooth rhythmic activation of the gastrocnemius and soleus muscle with weight added to precisely quantify relative loads. The angle of the footplate was adjusted (typically 0 to + 25 deg) and the workload was applied during flexion. The relaxation phase was unloaded to quantify skeletal muscle vasodilation. Exercise was performed with a duty cycle of two seconds contraction and four seconds relaxation for seven minutes. Participants were provided with real-time audio and instructor feedback to ensure correct contraction/relaxation timing. Heart rate and plantarflexor SmO_2_ were measured continuously through the protocol, whereas blood pressure and muscle blood flow (superficial femoral artery blood flow) were measured during a baseline period (~ 1 min) and the last 30 s of steady-state planter flexion. Representative data and equipment of experimental protocols are presented in Fig. 2.

### Measurements

#### Maximal voluntary contraction of plantarflexion

Isometric MVC of the plantarflexor muscles was determined by isometric measurement using a dynamometer (Con‐Trex MJ; CMV AG, Dubendorf, Switzerland). Each participant lay in the supine position, with an ankle angle of 0 degrees, and was asked to plantarflex against the dynamometer as hard as possible. Participants repeated the procedure three times, each separated by one minute of rest. If the three values were not within 10% difference, participants underwent additional trials. The greatest force output was taken as MVC.

#### Hemodynamics

Heart rate was continuously determined from an electrocardiogram. Systolic and diastolic arterial blood pressure were measured from the left arm using electro-sphygmomanometry (Tango; SunTechMedical Instruments Inc.). Mean arterial pressure was calculated using the formula: [(systolic blood pressure) + 2 × (diastolic blood pressure)]/3.

#### Superficial femoral artery diameter and blood velocity

Superficial femoral artery blood velocity and diameter were measured in duplex mode superficial to the femoral artery bifurcation using a 9-MHz linear-array Doppler probe (iE33; Philips, Netherlands) by duplex vascular sonography (iE33; Philips, the Netherlands). The time-averaged mean blood velocity was recorded at an insonation angle of 60° with the sample volume encompassing the entire vessel lumen. The ultrasound system was interfaced with custom software designed to continuously process the Doppler shift to measure the time-averaged mean velocity and edge-detection wall-tracking software for arterial diameter. The algorithm used to measure diameter has previously been validated (Coolbaugh et al. 2016) and used by our group to measure changes in femoral diameter associated with exercise (Amin et al. 2021a, b). All ultrasound measurements were conducted by the same experienced sonographer (K.M.), and once in position, a guide was adhered to the skin so that the same region of the vessel would be scanned in all trials. Femoral blood flow was determined through previously explained methods (Hansen et al. 2020; Amin et al. 2021a, b) and expressed in absolute terms as ml·min^−1^.

#### Skeletal muscle oxygenation by Near-Infrared Spectroscopy.

A commercial NIRS monitor (NIRO 200; Hamamatsu Photonics) continuously measured SmO_2_ (oxy-hemoglobin/total-hemoglobin × 100) at a sampling frequency of six Hz, with method of Spatial Resolved Spectroscopy. The sensors use three wavelengths (775, 810 and 850 nm) and contain two detectors located at a mean distance of 4 cm from the emitting source. A NIRS optode was placed on the gastrocnemius medialis (2/3 distance from the calcaneus and anterior fossa) and covered with an optically dense black material to minimize the intrusion of extraneous light.

### Data analysis

All cardiovascular variables were sampled at 400 Hz via an analog-to-digital converter (Powerlab; ADInstruments, Oxford, the UK) and displayed on LabChart (LabChart 8; AD Instruments, Oxford, the UK) and analyzed offline. The datasets generated and/or analyzed during the current study are available from the corresponding author on reasonable request.

#### Experimental protocol 1: reactive hyperemia and flow-medicated dilatation

Artery diameter and blood flow velocity were assessed in one minute before occlusion and continuously for three minutes following cuff deflation. The microvascular dilator response was assessed from the reactive hyperemia and quantified as the peak blood flow after cuff deflation (Iwamoto et al. 2018) and the upslope of a 10 s reperfusion window (slope 10 s) immediately following cuff deflation of SmO_2_ (Soares et al. 2020). In addition, blood flow area under the curve (BFAUC) between baseline and 100 s post-occlusion was calculated as Σ [1/2 (*xi* + 1 − *xi*) (*yi* + 1 − *yi*) + (*xi* + 1 − *xi*) (*yi* − *z*)], where *x* is time, *y* is blood flow and *z* is baseline blood flow (Iwamoto et al. 2018).

Regarding macrovascular dilator response, or endothelial function, FMD of the superficial femoral artery was quantified by calculating the percent increase in diameter from baseline measured before cuff inflation (one-minute average) to the peak 3-s average post-occlusion cuff release (Thijssen et al. 2011). Shear rates was calculated using equation: shear rate = 4 × (time-averaged mean blood velocity/diameter). The shear rate AUC was calculated from release of the occluding cuff until the peak diameter was achieved (Pyke et al. 2010); this variable was later used for calculate relative FMD (%FMD/shear rate AUC).

#### Experimental protocol 2: exercise-induced hyperemia

Superficial femoral artery blood flow was measured at rest and during steady-state exercise to evaluate exercise-induced changes in muscle blood flow, defined as the absolute and percent increase from rest to exercise.

### Statistical analyses

To determine the influence of leg position on the response to exercise or occlusion cuff release, independent paired t-test was used to compare the change score between leg positions (leg above the heart vs. leg below the heart). However, absolute muscle oxygenation during steady-state exercise was compared between leg positions. This is justified because baseline SmO_2_ was comparable between trails (*p* = 0.08) and the absolute degree of local hypoxia is the likely stimulus for training adaptations. For completeness, all data were also analyzed with two-way analysis of variance (leg position vs. time). Descriptive data for all conditions are presented in Tables 1 and 2. All statistical tests were two-sided and *p* ≤ 0.05 was regarded as statistically significant. All values are expressed as means ± standard deviation. All statistical analyses were performed using SPSS (Version 25, SPSS Inc., IBM, Chicago, IL) and Prism Graphpad (Version 8.4.2, GraphPad Software Inc., La Jolla, CA, USA).

**Table 1:** Hemodynamics at rest (baseline) and post-cuff release

	Leg above the heart		Leg below the heart		Main effect time	Main effect leg position	Interaction
	Baseline	Peak	Baseline	Peak			
Heart rate (bpm)	54 ± 8	54 ± 8	56 ± 9	53 ± 7	*P* = 0.95	*P* = 0.61	*P* = 0.49
Mean blood pressure (mmHg)	86 ± 6	No data	87 ± 8	No data			
Blood flow (mL·min^−1^)	88 ± 30	798 ± 231	93 ± 39	1348 ± 290**	*P* < 0.001	*P* < 0.001	*P* < 0.001
Diameter (cm)	0.58 ± 0.06	0.60 ± 0.07	0.57 ± 0.06	0.60 ± 0.07	*P* = 0.90	*P* = 0.24	*P* = 0.76
Share rate AUC × 10^3^ arbitrary units		11.9 ± 4.7		12.8 ± 3.8			
Absolute FMD (%)		3.0 ± 2.4		5.2 ± 2.1*			
Relative FMD		0.34 ± 0.37		0.43 ± 0.23			
SmO_2_ (%)	65 ± 3	75 ± 3	64 ± 5	77 ± 4	*P* < 0.001	*P* = 0.91	*P* = 0.31

^*^P < 0.05, **P < 0.01 compare with leg above the heart. Values are means ± SD

*FMD* flow-mediated dilation, *SmO*_*2*_ muscle oxygenation

**Table 2:** Hemodynamics at rest (baseline) and during steady-state planter flexion exercise

	Leg above the heart		Leg below the heart		Main effect time	Main effect leg position	Interaction
	Baseline	10%	Baseline	10%			
MVC (N)			108 ± 30				
10% MVC (kg)			18 ± 5				
Heart rate, bpm	55 ± 10	62 ± 11	54 ± 11	61 ± 10	*P* = 0.046	*P* = 0.86	*P* = 0.93
Mean blood pressure (mmHg)	87 ± 11	91 ± 11	85 ± 11	90 ± 13	*P* = 0.21	*P* = 0.54	*P* = 0.92
Blood flow (mL·min^−1^)	95 ± 31	285 ± 106	96 ± 24	374 ± 130**	*P* < 0.001	*P* = 0.09	*P* = 0.09
Diameter (cm)	0.57 ± 0.07	0.58 ± 0.07	0.57 ± 0.07	0.58 ± 0.06	*P* = 0.67	*P* = 0.95	*P* = 0.96
Shear rate (s^−1^)	48 ± 31	131 ± 58	45 ± 15	171 ± 83**	*P* < 0.001	*P* = 0.25	*P* = 0.18
SmO_2_ (%)	63 ± 5	41 ± 18	67 ± 7	67 ± 5**	*P* = 0.001	*P* < 0.001	*P* = 0.002

Values are means ± SD

^*^P < 0.05, **P < 0.01 compare with leg above the heart

*MVC* maximal voluntary contraction, *SmO*_*2*_ muscle oxygenation

## Results

### Experimental protocol 1: reactive hyperemia and flow-medicated dilatation

**Fig. 3:**
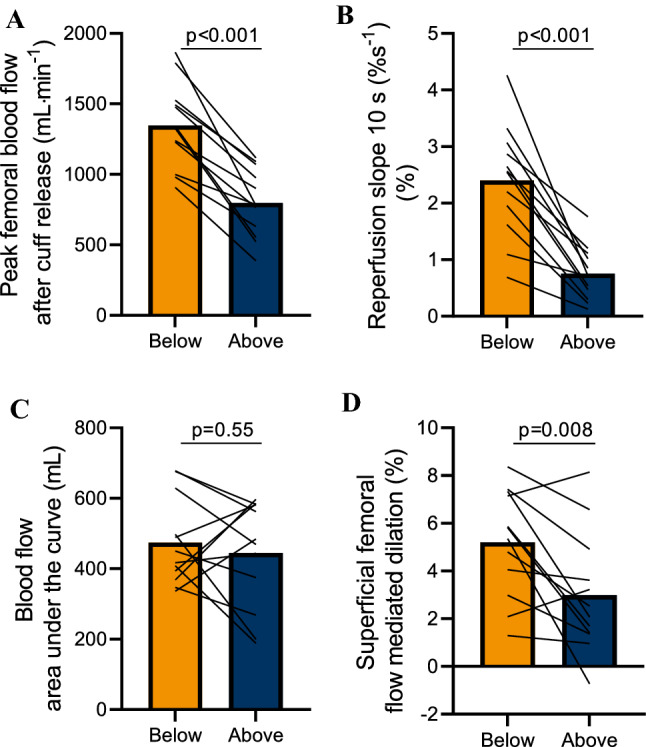
Micro- and macrovascular dilator response in the leg above the heart and leg below the heart positions

The reactive hyperemia (peak blood flow after cuff deflation) was lower with the leg positioned above the heart compared to below the heart (798 ± 231 mL·min^−1^ vs. 1348 ± 290 mL·min^−1^; *p* < 0.001) (Fig. 3a). Reperfusion slope 10 s was lower with leg above the heart position than with the leg below the heart position (0.75 ± 0.45% s^−1^ vs. 2.40 ± 0.94% s^−1^; *p* < 0.001) (Fig. 3B). Meanwhile, BFAUC at 100 s post-cuff occlusion was not altered by leg position (above the heart: 445 ± 147 mL vs. below the heart: 474 ± 118 mL; *p* = 0.55) (Fig. 3c). FMD was also smaller with leg above the heart position than leg below the heart position (3.0 ± 2.4% vs. 5.2 ± 2.1%; *p* = 0.008) (Fig. 3d) and driven predominantly by a lower shear rate stimulus, as the relative FMD (%FMD/shear rate AUC) was unaffected (above the heart: 0.34 ± 0.37 vs. below the heart: 0.43 ± 0.23; *p* = 0.34) (Table 1).

**Fig. 4:**
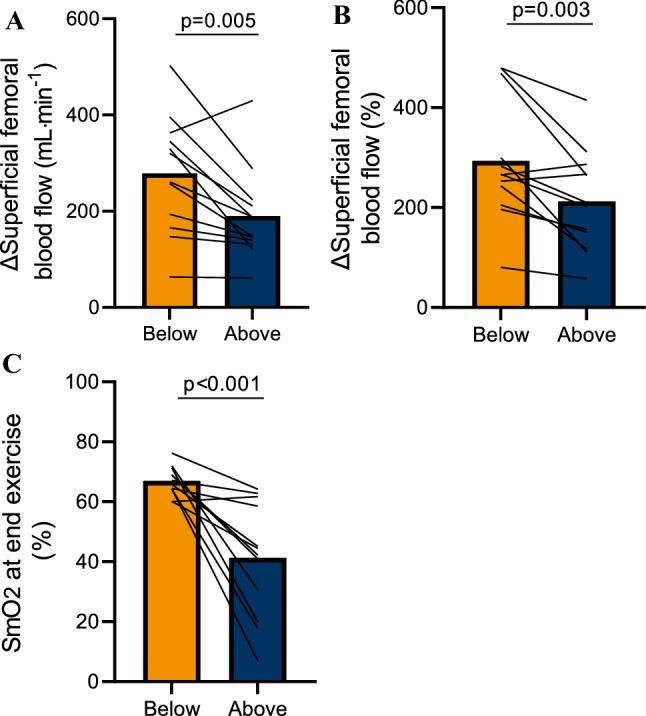
Exercise-induced hyperemia and muscle oxygenation (SmO2) in the leg above the heart and led below the heart positions

### Experimental protocol 2: Exercise-induced hyperemia

After 7-min rhythmical plantarflexion exercise at the same mechanical load (10% of MVC), heart rate and blood pressure increased during exercise and occurred similarly in both legs positions (*p* = 0.73 and *p* = 0.71, respectively) (Table 2). Exercise-induced hyperemia was lower with leg above the heart position compared to leg below the heart position (190 ± 91 mL·min^−1^ vs. 279 ± 117 mL·min^−1^; *p* = 0.005) (Fig. 4a), and the percent change in blood flow was also lower with leg above the heart position compared to leg below the heart position (212 ± 97% vs. 293 ± 118%; *p* = 0.003) (Fig. 4b).

### Experimental protocol 2: Skeletal muscle oxygenation by Near-Infrared Spectroscopy

Baseline SmO_2_ was comparable in both positions (63 ± 5% vs. 67 ± 7%; *p* = 0.08). During exercise, SmO_2_ remained at the same level as a baseline with leg below the heart position (Table 2). At the end of the exercise, SmO_2_ was lower with leg above the heart position compared to leg below the heart position (41 ± 18% vs. 67 ± 5%; *p* < 0.001) (Fig. 4d).

## Discussion

The major finding of the present study is that positioning the legs above the heart results in attenuated onset microvascular dilator response and exercise-induced hyperemia that coincided with a lower SmO_2_ during low-intensity plantarflexion exercise.

SmO_2_ represents the balance between oxygen delivery and oxygen utilization (Ferrari et al. 2011). In the present study, the same mechanical load was utilized for all plantarflexion exercises. Thus, we assume that oxygen demand was the same and did not affect the difference in SmO_2_ in the two leg positions (leg above and below the heart level positions). The observed reduction in skeletal muscle blood flow during the leg above the heart position would be associated with reduced oxygen delivery and thus may contribute to the lower SmO_2_. A major mechanism to explain the reduction in blood flow with leg above the heart position is the reduction in hydrostatic pressure. A lower perfusion pressure gradient occurs in the lower limbs due to a lower hydrostatic pressure (Rowell 1974; Laughlin and Joyner 2003). Moreover, the difference in muscle pump effects should also be related to our observations; the effect of muscle pump on the arteriovenous pressure gradient for limb blood flow is dependent on the available venous volume to empty (Pollack and Wood 1949; Folkow et al. 1970). Thus, the effect of the muscle pump is smaller in the leg above the heart position compared to the leg below the heart position (Bevegard and Lodin 1962; Tschakovsky et al. 1996). In addition, as hydrostatic pressure is one of the main stimulators of the vascular endothelium (Prystopiuk et al. 2018), an environment with a reduced hydrostatic pressure (i.e., leg above the heart position) should impair the ability of the vasculature to vasodilate. In our study, during light planterflexion exercise, peak blood flow after cuff deflation, reperfusion slope 10 s, and FMD were attenuated in the leg above the heart position with the consequence of a lower femoral blood flow response. Considering BFAUC after cuff release was comparable between two leg’s positions, leg position can especially affect acute phase of vasodilation response. While we observed that the leg above the heart position caused reduced conduit artery endothelial response (i.e., reduced FMD), it is important to note that the attenuation in blood flow thru the femoral artery also reduces endothelial shear stress, which is a major factor for conduit artery dilation (Pyke and Tschakovsky 2005). Indeed, when corrected for shear rate, femoral artery dilation was comparable independent of leg position. An alternative explanation for the lower SmO_2_ was that oxygen extraction increased to meet the same metabolic demand under conditions of reduced oxygen delivery. Future studies directly measuring oxygen delivery and arterial–venous oxygen difference are required to separate these factors.

Studies have shown that hypoxia, as a result of BFR, can induce alterations on downstream mechanisms resulting in an augmentation of muscle hypertrophy (Scott et al. 2014). For example, hypoxia causes an earlier shift to anaerobic metabolism and increases lactate (Kon et al. 2010), stimulating the overall release of hormones (Gordon et al. 1994; Goto et al. 2005), which in turn promote muscular hypertrophy (Florini et al. 1996; McCall et al. 1999). Numerous investigations have shown that BFR training at a low-intensity promoted elevations in hypertrophy and strength (Takarada et al. 2002, 2004). However, BFR training, which requires external pressure to occlude blood flow, has several limitations: 1) monitoring occlusion pressure (Sumide et al. 2009; Scott et al. 2014), and 2) the complications of the technique. Typical complications of the cuff or elastic wraps are petechial hemorrhage beneath the skin, chills, numbness, and dizziness (Scott et al. 2014). Moreover, several studies reported a greater pain score during low-intensity RT with BFR compared to those without BFR (Wernbom et al. 2006; Loenneke et al. 2011).

Our results suggest that positioning legs above the heart position, which is a safe and simple technique, can result in localized hypoxia and therefore may be an effective training strategy to promote skeletal muscle adaptations. The implications of positioning legs above heart level would allow for similar skeletal muscle and vascular adaptions within populations confined to bedrest, aging, or otherwise, as load-bearing activities and/or exercises. It is said that 30–60% of elderly patients lose some independence in basic activities of daily living in the course of a single hospital stay (Lafont et al. 2011). This is mainly because patients are often incapable of applying high mechanical loads because of their compromised strength and joint stability. Thus, the use of low-intensity RT with leg above the heart position could overcome this issue.

To apply low-intensity RT with the leg above the heart position as a training method, further studies are warranted. It is interesting to compare the effect of BFR and leg above the heart position on low-intensity RT. One potential study topic is a comparison of SmO_2_ reduction during exercise with BFR and leg above the heart position. A previous study using plantarflexion exercise with BFR at 30% of MVC for four minutes (30 repetitions/min) showed SmO_2_ of medial gastrocnemius decreased from ~ 65 to ~ 20% after two minutes from exercise start and kept ~ 20% till exercise ended (Yanagisawa and Sanomura 2017). With leg above the heart position in our study, baseline SmO_2_ was 63 ± 5% and decreased to 41 ± 19% after 1 min from exercise and kept around 40% till exercise ended. Although a lighter exercise load was imposed, a smaller drop of SmO_2_ was observed with the leg above the heart position in our study compared to plantarflexion exercise with BFR at 30% of MVC in a previous study. To compare the effect of leg position and BFR on muscle oxygenation during exercise, a study using the same exercise protocol is needed. Another potential study topic is a comparison of exertion time between exercise with BFR and leg above the heart position. To assess this point, a further study needs to impose higher intensity exercise or longer exercise time compared to our study since participants did not reach task failure due to low-intensity exercise for seven minutes in our study.

## Limitations

Firstly, our study was conducted in a healthy and young adult population. We acknowledge that the practical application of contracting muscle position during supine exercise should be targeted toward elderly and/or clinical populations who are unable to exercise in the upright position. Future studies should focus on such groups to determine contracting muscle position would induce similar results. Second, although maximal dilator response was assessed, true exercise was only performed at a light intensity where SmO_2_ is maintained at baseline values. Higher intensity exercise would be an obvious extension if technical limitation of measuring limb blood flow and shear can be overcome. Third, any link between lower muscle oxygenation and greater training adaptations are purely speculative and careful training studies are needed to provide conclusive evidence. Lastly, we did not control the female menstrual cycle. Endothelial function can be altered by hormone state. Since the tests were conducted on the same day and the endothelial response in the two leg positions of each participant was compared, it was considered that the hormonal status would not strongly influence the results.

## Conclusion

To conclude, when the leg is above heart level, exercise-induced hyperemia and vascular dilator response are reduced, resulting in a lower muscle oxygenation despite low-intensity plantarflexion exercise. Therefore, RT in the leg above the heart position may be an effective exercise method to combat physical deconditioning when persons are unable to lift heavy mechanical loads.

## Data Availability

The datasets generated and/or analyzed during the current study are available from the corresponding author on reasonable request.

## Abbreviations

BFR: Blood flow restriction
BFAUC: Blood flow area under the curve
FMD: Flow-mediated dilation
MVC: Maximum voluntary contraction
NIRS: Near-infrared spectroscopy
SmO_2_: Muscle oxygenation
